# Optimizing Poultry Growth and Meat Quality: Effects of Guanidinoacetic Acid Supplementation in Yellow-Feathered Broilers

**DOI:** 10.3390/vetsci12060551

**Published:** 2025-06-05

**Authors:** Jian Xiao, Lifen Wang, Yuguang Chen, Kai Xiao

**Affiliations:** Animal Nutritional Genome and Germplasm Innovation Research Center, College of Animal Science and Technology, Hunan Agricultural University, Changsha 410128, China; xjxj0814@163.com (J.X.); wanglifen1118@163.com (L.W.)

**Keywords:** guanidinoacetic acid, yellow-feathered broilers, growth performance, slaughter performance, meat quality

## Abstract

This study investigated the effects of dietary guanidinoacetic acid (GAA) supplementation on growth performance, carcass traits, meat quality, intestinal morphology, and cecal microbiota composition in yellow-feathered broilers. A total of 360 one-day-old chicks were randomly assigned to five groups with diets containing 0 (control), 300 mg/kg, 600 mg/kg, 900 mg/kg, and 1200 mg/kg GAA. In the initial growth phase, GAA supplementation increased average daily gain (ADG) by 13.01%, 8.97%, and 12.95% for the 300, 600, and 900 mg/kg groups, respectively, and reduced the feed conversion ratio (FCR), though these changes were not statistically significant (*p* > 0.05). Higher GAA dosages (900 and 1200 mg/kg) improved post-slaughter pH levels in breast muscle, indicating better meat quality, and significantly enhanced intestinal barrier function by increasing villus height and the villus-to-crypt ratio. However, 1200 mg/kg GAA led to a significant increase in serum ALT levels, raising concerns about potential liver stress. Overall, 900 mg/kg GAA supplementation demonstrated the most beneficial effects across various parameters, suggesting it as an optimal dosage to enhance production performance and meat quality in yellow-feathered broilers. Future studies are encouraged to investigate its long-term safety and underlying mechanisms.

## Simple Summary

This study evaluated the effects of dietary guanidinoacetic acid (GAA) supplementation on the growth performance, intestinal health, and meat quality of yellow-feathered broilers. Among the tested dosages, 900 mg/kg GAA demonstrated the most comprehensive benefits, improving feed utilization efficiency, enhancing intestinal health, and producing higher-quality meat. However, extremely high doses (1200 mg/kg) led to a significant increase in a liver function marker, suggesting potential risks to liver health. These findings highlight GAA’s potential as a safe and effective dietary additive at appropriate levels, offering valuable insights for optimizing poultry production and addressing challenges in sustainable livestock systems. Future studies should further explore its long-term effects and underlying mechanisms.

## 1. Introduction

In recent years, advances in genetic breeding, feed nutrition research, and large-scale farming technologies have significantly improved the growth performance of broilers. However, these improvements have been accompanied by a noticeable decline in meat quality, alongside an increasing incidence of meat quality-related conditions such as white striping, spaghetti meat, and woody breast [1,2,3,4,5,6,7]. To address this challenge, slow-growing breeds known for their superior meat quality compared to traditional commercial breeds have been adopted, providing a potential solution to balance growth performance and meat quality. Concurrently, economic development has shifted dietary preferences from simply “eating enough” to “eating well”. This transition underscores the poultry industry’s ultimate goal of enhancing meat quality while maintaining high production levels. Creatine, a nitrogen-containing amino acid and energy buffer, plays a crucial role in muscle energy metabolism. Through creatine kinase, it forms phosphorylated creatine, which participates in the adenosine triphosphate (ATP) cycle by rapidly donating phosphate groups to regenerate ATP during energy shortages. Skeletal muscle contains approximately 95% of the body’s creatine pool [8], yet about 1.7% of creatine and phosphorylated creatine are irreversibly converted to creatinine daily and excreted, necessitating continuous dietary supplementation [9]. Traditionally, creatine supplementation relied on animal-derived ingredients such as fishmeal, which are costly and present biosecurity concerns. With the increasing adoption of all-plant diets lacking sufficient creatine, identifying effective feed additives to supply this essential compound has become critical.

Yellow-feathered broilers hold a prominent position in poultry farming, particularly in China, due to their tender, flavorful meat highly valued as a premium food ingredient. Beyond superior meat quality, these broilers offer significant economic advantages owing to their higher market price and shorter rearing cycles, which reduce farming risks. Their strong adaptability to various environments and feed resources lowers production costs, while their rich protein and micronutrient content aligns with modern healthy dietary trends. Moreover, the farming of yellow-feathered broilers supports related industries, promotes rural economic development, and offers potential for genetic improvement and enhanced disease resistance, making them indispensable in the poultry sector.

Guanidinoacetic acid (GAA), a natural amino acid derivative and direct precursor to creatine, has demonstrated efficacy in enhancing energy metabolism and improving growth performance and muscle development in broiler chickens [10,11,12,13,14,15,16,17,18,19,20,21,22,23]. Studies have shown that the dietary supplementation of GAA at levels between 0.6 and 1.2 g/kg significantly improves growth performance metrics and breast muscle yield [10,11,12,13]. Additionally, GAA enhances feed conversion rates and reduces energy requirements for weight gain [14]. Supplementation has also been linked to increased average daily gain, improved feed efficiency, higher survival rates, and elevated European broiler index scores [16]. Despite these findings, limited research exists on the application of GAA in yellow-feathered broilers. Mechanistically, GAA promotes the secretion of gamma-aminobutyric acid (GABA), stimulating the hypothalamus to release growth hormone-releasing hormone, which in turn promotes the pituitary secretion of growth hormone, facilitating animal growth [24]. Furthermore, GAA has been shown to mitigate muscle disorders [25], and its superior thermal stability compared to creatine during feed processing minimizes nutrient loss, highlighting its potential as an effective feed additive.

Given this background, the present study aims to evaluate the effects of GAA supplementation on growth performance, meat quality, and intestinal microbiota in yellow-feathered broilers. We hypothesize that supplementation levels of 300 mg/kg and 600 mg/kg will significantly enhance average daily gain and feed conversion rates compared to the control and higher supplementation levels. Additionally, we expect improvements in meat quality parameters, including a reduced pH of the pectoralis major muscle, enhanced water retention capacity, and improved color, aligning with consumer preferences. We also anticipate that GAA will positively modulate intestinal microbiota diversity and abundance, promoting gut health and nutrient absorption. Through this investigation, we seek to provide scientific evidence supporting the application of GAA in yellow-feathered broiler production, ultimately improving growth efficiency and meat quality.

## 2. Materials and Methods

The experimental protocol was approved by the Animal Care and Use Committee of Hunan Agricultural University (permit number: CACAHU 2019-0312) according to the Chinese Guidelines for Animal Welfare.

### 2.1. Animals, Diets, and Experimental Design

The guanidinoacetic acid (GAA) utilized in this study, supplied by Sichuan Dukang Biotechnology Co., Ltd., Dazhou, China, had a purity of 99%. Arginine, a conditionally essential amino acid, is pivotal in muscle metabolism and contributes to the synthesis of creatine. Given that GAA is a direct precursor to creatine, the supplementation of GAA in the diet is expected to enhance muscle growth and overall performance in poultry. This study aimed to investigate the effects of varying GAA levels on the growth performance and feed efficiency of the broiler chicks.

1. CON Group: Fed with the basal diet.

2. GAA300 Group: Basal diet supplemented with 300 mg/kg GAA.

3. GAA600 Group: Basal diet supplemented with 600 mg/kg GAA.

4. GAA900 Group: Basal diet supplemented with 900 mg/kg GAA.

5. GAA1200 Group: Basal diet supplemented with 1200 mg/kg GAA.

Each group comprised 6 replicates, with 12 chicks per replicate. The experiment lasted for a total of 60 days and was divided into two phases: the early phase (1 to 28 days of age) and the late phase (29 to 60 days of age). Fast-growing male yellow-feathered broilers were selected for the trial to minimize growth variations caused by sex hormones, especially estrogen, which can result in differing growth performance between hens and cockerels. The one-day-old chicks had a closely matched average body weight of 35.8 g.

By carefully controlling the diet and standardizing the initial conditions for all groups, this experimental design aims to evaluate the effects of varying levels of guanidinoacetic acid supplementation on the growth performance and overall health of broilers over a 60-day period.

The basal diets were formulated based on the nutritional requirements of broiler chickens as outlined in the NY/T 33-2004 Chicken Feeding Standard, which serves as a widely accepted guideline for poultry nutrition in China. The diets were designed to meet the birds’ needs during the early (1–28 days) and later (29–60 days) growth phases. The detailed composition and calculated nutrient levels are presented in Table 1.

To ensure the homogeneity of small-scale experimental diets, major and minor feed ingredients (e.g., limestone, dicalcium phosphate, salt) and micro-nutrients were pre-mixed into a premix (Table 2), which was incorporated into the complete diet at a level of 4% (40 kg/ton). This approach aligns with experimental feed preparation practices, allowing for precise nutrient supplementation. Our feed formulation was carried out at the Pursuit Feed Factory of Hunan Agricultural University.

### 2.2. Housing, Feeding, and Sample Collection

The experiment was conducted at the Hunan Poultry Safety Production Engineering Technology Research Center, Hunan Agricultural University. One week prior to the commencement of the study, the poultry house was fumigated with a combination of potassium permanganate and formalin, followed by three days of ventilation to ensure a sterile environment.

The broilers were housed in a fully enclosed, multi-tier cage system, with three levels (upper, middle, and lower). Each cage measured 1.2 m in length, 1.6 m in width, and 0.46 m in height. Each experimental group was distributed across all three levels, with replicate cages randomly assigned to minimize potential positional effects. The cages were equipped with nipple drinkers to ensure a consistent water supply. Feeding occurred three times daily at 08:00, 14:00, and 20:00. Throughout the experiment, feed and water were provided ad libitum.

To maintain optimal conditions, the poultry house was equipped with evaporative cooling pads and exhaust fans on both sides, as well as thermometers and hygrometers to monitor temperature and humidity. A boiler system was used for heating, with carbon being added every three hours to maintain a consistent temperature.

Lighting Schedule: Continuous lighting was provided for the first 15 days (24 h per day). After 15 days, the lighting schedule was adjusted to include one hour of darkness per day, with lights turned off at 19:00.

Immunization: The broilers were vaccinated according to standard immunization protocols.

General Management: Routine management practices were followed in accordance with conventional poultry rearing procedures.

Health Monitoring: Daily observations were made to assess the health status of the flock.

By implementing rigorous pre-experimental sanitization and maintaining controlled environmental conditions, this study aimed to minimize extraneous variables, thus ensuring that any observed effects can be attributed to the experimental treatments. The careful randomization of replicates and adherence to standardized management protocols further enhance the reliability of the findings.

### 2.3. Chemical Analysis and Digestibility Calculation

#### 2.3.1. Growth Performance Indicators

During the experimental period, meticulous daily records were maintained for each replicate, documenting feed intake, feed leftovers, feed loss, and the number of culled or deceased chickens. Weights were measured at 1, 28, and 60 days of age, with morning measurements conducted at 7 am on an empty stomach. Feed was withdrawn at midnight on the days preceding the 28th and 60th measurements. The rationale for selecting these particular time points is due to the dietary changes that occur during the trial. From 1 to 28 days of age, the chicks were fed a starter diet specifically formulated for young broilers. Following this period, from 29 to 60 days of age, they were transitioned to a grower diet designed to support growth in the later stages of development. By assessing the weights at these critical stages, we aimed to capture the impact of the dietary transition on growth performance effectively. This approach allowed us to evaluate weight gain during the starter phase, as well as the subsequent growth response during the grower phase. Parameters such as average daily feed intake (ADFI), average daily gain (ADG), and feed-to-gain ratio (F/G) were calculated, while the age at death and weight of deceased chickens were documented.

#### 2.3.2. Serum Biochemical Parameter Measurement

On the 60th day of the experiment, one chicken with body weight close to the average was selected from each replicate for jugular vein blood sampling. Blood samples of 5 mL were collected into tubes (two-thirds full), inclined at 30° for 30 min, and then centrifuged at 3000 r/min for 10 min to separate the serum. The upper layer of serum was collected into centrifuge tubes and stored at −80 °C. Serum levels of alanine aminotransferase (ALT), glucose (GLU), triglycerides (TG), low-density lipoprotein (LDL-C), and high-density lipoprotein (HDL-C) were measured using Shanghai Kehua Bio-reagent kits (ALT: C009-3-1, GLU: F006-1-1, TG: A110-1-1, LDL-C: SP00287, HDL-C: SP00286) and the Excellent ZY-450 automatic biochemical analyzer (Shanghai, China).

#### 2.3.3. Intestinal Morphology Observation

On the 28th and 60th days of the experiment, one chicken with body weight close to the group average was selected from each replicate. After exsanguination via the jugular vein and rapid dissection, the intestines were removed and divided into segments. Mid-segments of the jejunum and cecum (1–2 cm each) were rinsed with physiological saline to remove chyme, dried with clean filter paper, and fixed in 4% paraformaldehyde solution. Fixed samples were then dehydrated, cleared, embedded in paraffin, trimmed, sectioned, mounted, and stained with routine hematoxylin–eosin (HE) to prepare paraffin sections. Multiple random non-continuous fields of view were observed under a microscope, and representative images were captured. Observations and measurements—including villus height, crypt depth in the jejunum and cecum, and the villus height to crypt depth ratio (V/C)—were performed using the MSHOT ML31 microscopic imaging system (Guangzhou, China) and its IMAGEEX software (version V9.0, software copyright number 2009SR034762) from Guangzhou Ming-Mei Technology Co., Ltd., Guangzhou, China [26,27].

#### 2.3.4. Measurement of Lactate and Glycogen in Muscle Tissue Samples

On the 28th and 60th days of the experiment, one chicken with body weight close to the average was randomly selected from each replicate group. Following exsanguination via the carotid artery and immediate dissection, breast muscle samples were collected from consistent anatomical locations, frozen in liquid nitrogen, and stored at −80 °C. Lactate (LD), glycogen (GLU), and total protein (TP) contents in the breast muscle were measured using assay kits from Nanjing Jiancheng Bioengineering Institute [28].

#### 2.3.5. Paraffin Embedding and HE Staining

On the 28th and 60th days of the experiment, one chicken with body weight close to the average was selected from each replicate group. After exsanguination via the jugular vein, breast muscle samples were collected from the same anatomical location and fixed in 4% paraformaldehyde solution. The fixed samples underwent dehydration, clearing, paraffin embedding, trimming, sectioning, spreading, and routine hematoxylin–eosin (HE) staining to create paraffin sections. Multiple random non-continuous fields of view were observed with a microscope, representative fields were photographed, and muscle fiber diameter and density were measured using ImageJ (v.1.54) software.

#### 2.3.6. Slaughter Performance

On the morning of day 60 of the experiment, one chicken with body weight close to the average was randomly selected from each replicate group. After weighing on an empty stomach, the chicken was exsanguinated via the carotid artery and slaughtered. The weights of the semi-eviscerated carcass, fully eviscerated carcass, breast muscles, leg muscles, and abdominal fat were measured. Various slaughter performance metrics were calculated following the methods detailed in the “Poultry Production Performance Terms and Measurement Statistics Methods” (NY/T 823—2020).

Calculation methods:

Slaughter Rate (%) = (Carcass Weight/Pre-Slaughter Weight) × 100%

Semi-Eviscerated Rate (%) = (Semi-Eviscerated Weight/Pre-Slaughter Weight) × 100%

Fully Eviscerated Rate (%) = (Fully Eviscerated Weight/Pre-Slaughter Weight) × 100%

Breast Muscle Rate (%) = (Weight of Both Breast Muscles/Fully Eviscerated Weight) × 100%

Leg Muscle Rate (%) = (Weight of Both Leg Muscles/Fully Eviscerated Weight) × 100%

Abdominal Fat Rate (%) = (Abdominal Fat Weight/(Fully Eviscerated Weight + Abdominal Fat Weight)) × 100%

Meat Quality Indicators

On the morning of the 60th day of the experiment, after the broiler chickens were slaughtered, a whole breast muscle from the same side was taken to measure various meat quality indicators, such as drip loss, pressure loss, pH value, meat color, and shear force. The specific methods are as follows. 

Drip Loss:

Within 45 min post-slaughter, muscle samples were trimmed into 2 × 2 × 1 cm^3^ pieces using surgical scissors, and their weights were recorded as D1. A wire was passed through the center of an inverted plastic cup, and one end of the wire was hooked to the muscle sample, maintaining it in a vertical orientation. This setup was placed inside the plastic cup, sealed with a plastic bag, and stored in a 4 °C refrigerator for 24 h. The sample was then weighed again (D2) after gently blotting surface fluids with clean filter paper. The drip loss rate was calculated using the following formula:

Drip Loss Rate (%) = ((D1 − D2))/D1 × 100%

Pressure Loss:

Post-slaughter, the skin, fat, and connective tissues were removed from the breast muscle. Cylindrical samples with a diameter of 2.5 cm and a thickness of 1 cm were cut perpendicular to the muscle fibers and weighed (S1). The round sample was wrapped in two layers of medical gauze, placed between several layers of qualitative filter paper, and compressed between iron plates using a modified hydraulic press at 35 kg pressure (96.2 on the hydraulic gauge) for 5 min. After removing the filter paper and gauze, the sample was weighed again (S2). The pressure loss rate was calculated as

Pressure Loss Rate (%) = ((S1 − S2))/S1 × 100%

pH Value:

Using a portable pH meter, the pH value was measured 45 min post-slaughter. The calibrated pH meter probe was inserted into the upper middle part of the same side of the breast muscle to ensure full contact with the tissue fluids. The reading was recorded once stable (15–20 s). Each sample was measured three times, and the arithmetic mean was taken as the 45 min post-mortem pH value. The samples were then sealed in zip-lock bags and stored in a 4 °C refrigerator. After 24 h, the measurements were repeated three times, and the arithmetic mean was recorded as the 24 h post-mortem pH value.

Meat Color:

Within one hour post-slaughter, the breast muscle was laid flat on a clean tray (ensuring a smooth surface). A calibrated colorimeter was placed vertically on the muscle surface, avoiding connective tissue, blood clots, and intramuscular fat. The device was held flush against the muscle surface (without light leakage). The lightness (L*), redness (a*), and yellowness (b*) values of the meat color were measured and recorded. Each sample was measured three times, and the arithmetic mean served as the final result.

Shear Force:

At the experiment’s conclusion, breast muscle samples (with consistent muscle fiber direction and thickness greater than 2.5 cm) were collected. Six cylindrical muscle samples with a diameter of 1.30 cm were obtained along the muscle fiber direction and placed horizontally at the shear apparatus’s knife edge for cutting. The shear force values were recorded.

#### 2.3.7. Statistical Analysis

The experimental data were systematically organized and subjected to statistical analysis using Excel 2019 and SPSS version 25.0. A one-way analysis of variance (ANOVA) was performed to evaluate the differences among the treatment groups, allowing us to determine if the guanidinoacetic acid supplementation significantly affected the measured parameters. To discern specific group differences following the ANOVA, Duncan’s multiple-range test was applied as a post hoc analysis, facilitating a thorough comparison of means among the groups.

In addition to standard significance testing, we examined trends with *p*-values between 0.05 and 0.1 to provide insights into potential patterns that might warrant further investigation. All values presented in the tables represent the means ± standard error (SE). We established a significance threshold of *p* < 0.05 to denote statistical significance, with *p* < 0.01 indicative of highly significant differences. Non-significant trends were cautiously included when they offered valuable context to the results, enhancing the overall narrative of our findings.

To explore the effects of varying levels of guanidinoacetic acid supplementation on growth performance and other metrics, we employed both linear and quadratic regression analyses. The linear regression model assessed the relationship between the dosage of guanidinoacetic acid and response variables such as average daily gain (ADG) and average daily feed intake (ADFI). This analysis allowed us to establish whether there is a direct proportional relationship between these variables.

We also implemented a quadratic regression analysis to investigate potential non-linear relationships, allowing us to identify any diminishing returns or optimal dosage levels associated with guanidinoacetic acid supplementation. This model provided insights into the peak effects and highlighted how varying dosages impact the performance metrics in a more complex manner.

The outputs from both regression analyses were evaluated for goodness of fit through R^2^ values, with higher R^2^ values indicating a better fit of the model to the data. The significance of the regression coefficients was examined with corresponding *p*-values to ensure robust insights into the relationships observed in our study.

## 3. Results

### 3.1. Influence of Guanidinoacetic Acid on the Growth Performance of Yellow-Feathered Broiler Chickens

**Figure 1:**
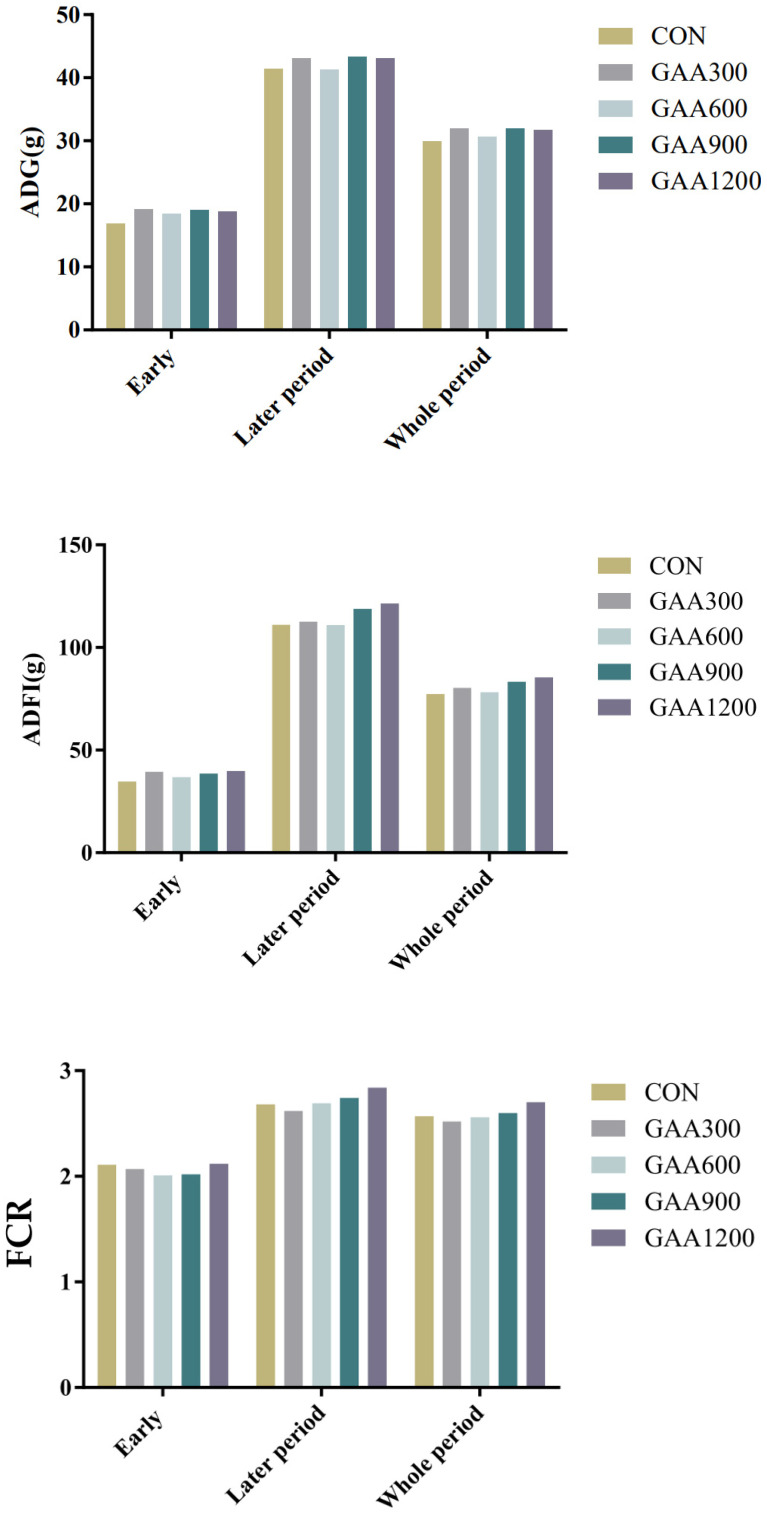
The effect of guanidinoacetic acid supplementation on growth performance during the entire growth period of yellow-feathered broiler chickens.

As shown in Figure 1, this study evaluated the effects of dietary GAA supplementation at different dosages on the growth performance of yellow-feathered broilers. No statistically significant differences were observed among the experimental groups in average daily gain (ADG), average daily feed intake (ADFI), or feed conversion ratio (FCR) during the early phase, later phase, or the whole experimental period (*p* > 0.05). However, numerical trends were identified in FCR, suggesting potential improvements in feed efficiency at specific GAA dosages. During the early phase, broilers in the GAA600 group demonstrated the lowest FCR (2.01), followed closely by the GAA900 group (2.02), compared to the control group (2.11). This indicates a potential enhancement in feed utilization efficiency at moderate GAA supplementation levels. In the later phase, the GAA300 group had the lowest FCR (2.62) compared to the other treatment groups, while the GAA1200 group exhibited the highest FCR (2.84), suggesting that higher GAA dosages may not further improve feed efficiency in this phase. Over the entire experimental period, the GAA300 group maintained the lowest FCR (2.52) among all treatment groups, slightly lower than the control group (2.57). Conversely, the GAA1200 group showed the highest FCR (2.70), indicating that excessive dosages may compromise feed utilization. These numerical trends suggest that moderate GAA supplementation levels (300–600 mg/kg) may improve feed efficiency, particularly during the early growth phase.

### 3.2. Influence of Guanidinoacetic Acid on the Slaughter Performance of Yellow-Feathered Broiler Chickens

**Figure 2:**
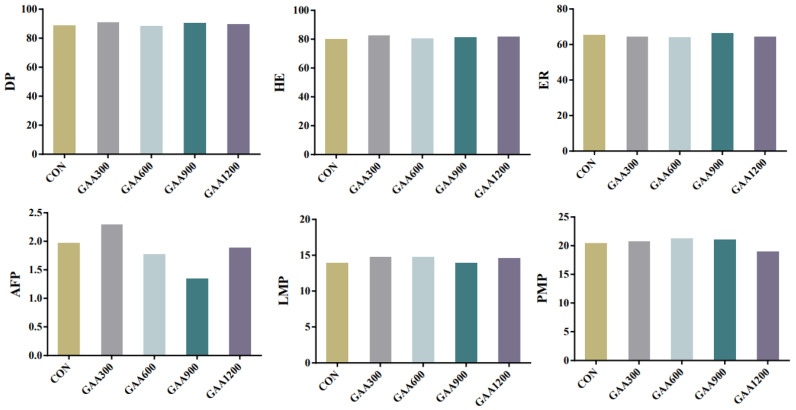
Slaughter Performance of Yellow-Feathered Broiler Chickens with Varying Doses of Guanidinoacetic Acid.

As shown in Figure 2, no statistically significant differences (*p* > 0.05) were observed in dressing percentage, evisceration rate, abdominal fat percentage, or muscle percentages among the groups. However, the dressing percentage and evisceration rate tended to be higher in the GAA900 group (90.48% and 66.32%, respectively), and this group also showed the lowest abdominal fat percentage (1.35%). Additionally, the GAA600 group achieved the highest leg and pectoral muscle percentages (14.76% and 21.13%, respectively). These findings suggest that moderate-to-high doses of GAA supplementation (600–900 mg/kg) may improve certain carcass traits in yellow-feathered broilers, warranting further investigation.

### 3.3. Influence of Guanidinoacetic Acid on the Quality of Breast Meat in Yellow-Feathered Broiler Chickens

**Figure 3:**
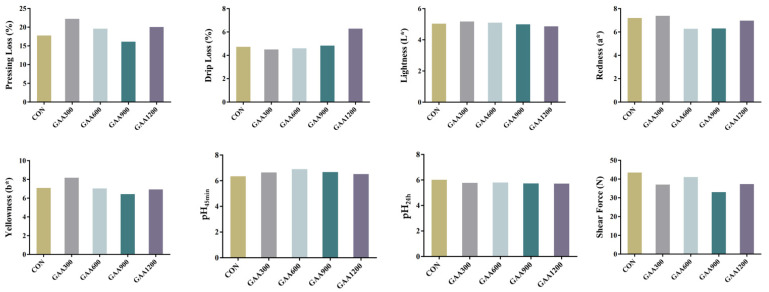
Effects of Various Doses of Guanidinoacetic Acid on the Meat Quality of Yellow-Feathered Broiler Chickens.

According to Figure 3, guanidinoacetic acid (GAA) supplementation significantly influenced several meat quality traits in yellow-feathered broilers. For water-holding capacity, the GAA300 group had the lowest drip loss (4.50%), significantly lower than the control group (*p* < 0.05), indicating improved water retention. Additionally, pressing loss was the lowest in the GAA900 group (16.14%), further demonstrating enhanced water holding capacity with GAA supplementation. However, excessive supplementation (GAA1200) increased drip loss to 6.28% (*p* < 0.05), suggesting a negative effect at higher doses. Regarding meat color, no significant differences were observed in lightness (L*) or redness (a*) among groups, but the GAA300 group showed a slight increase in yellowness (b*), potentially improving meat appearance. In terms of pH values, the GAA600 group achieved the highest pH45 min (6.89, *p* < 0.01), reflecting a better acid–base balance post-slaughter. Changes in pH after 24 h were minimal across treatments. For meat tenderness, the GAA900 group had the lowest shear force value (33.07 N), indicating a trend toward more tender meat, although this difference was not statistically significant. Overall, moderate GAA supplementation (300–900 mg/kg) effectively improved water holding capacity, meat tenderness, and some aspects of meat color. However, excessive supplementation (e.g., 1200 mg/kg) may negatively affect water retention. These findings suggest that GAA supplementation at appropriate levels has promising potential to enhance meat quality in broilers.

### 3.4. Impact of Guanidinoacetic Acid on Serum Biochemical Parameters of Yellow-Feathered Broiler Chickens

Table 3 summarizes the effects of dietary guanidinoacetic acid (GAA) supplementation on serum biochemical parameters at different growth stages (28 and 60 days). In the early stage (28 days), GAA supplementation did not significantly affect serum alanine aminotransferase (ALT), glucose (GLU), triglycerides (TG), high-density lipoprotein (HDL), or low-density lipoprotein (LDL) levels compared with the control group (*p* > 0.05). These results indicate that early-stage GAA supplementation does not impair liver function or disrupt the metabolic stability of broilers. In the late stage (60 days), dietary GAA supplementation significantly influenced serum ALT levels (*p* < 0.05). The ALT level in the GAA1200 group (3.33 U/L) was significantly higher than that in the control and other treatment groups, suggesting potential risks of metabolic stress or liver burden at higher doses. By contrast, ALT levels in the GAA300, GAA600, and GAA900 groups (1.83–3.00 U/L) remained comparable to the control group, indicating no adverse effects on liver function at these doses. Similarly, other parameters, including GLU, TG, HDL, and LDL, showed no significant changes across treatments (*p* > 0.05), reflecting stable metabolic profiles with GAA supplementation. Notably, the GAA900 group maintained favorable serum biochemical parameters without significant deviations, making it a potentially optimal dosage to balance metabolic stability and performance. However, excessive GAA supplementation (e.g., 1200 mg/kg) should be approached with caution due to the observed increase in ALT levels.

### 3.5. The Impact of Guanidinoacetic Acid on Lactate and Glycogen Levels in the Pectoral Muscles of Yellow-Feathered Broilers

As shown in Table 4, the effects of dietary supplementation with guanidinoacetic acid (GAA) on the lactate (LD) and glycogen (GLU) content in the pectoral muscles of yellow-feathered broilers are presented in Table 4. Measurements were conducted at both 28 days (early phase) and 60 days (later phase). At 28 days, there were no significant differences in LD and GLU levels across treatment groups (*p* > 0.05). Although slight numerical variations were observed (e.g., LD ranged from 0.56 to 0.83 mmol/g, while GLU ranged from 0.57 to 1.12 mg/g), the differences did not reach statistical significance (*p* = 0.451). Similarly, at 60 days, GAA supplementation had no significant effect on LD levels (*p* = 0.786) or GLU levels (*p* = 0.948), with values remaining stable across all groups. Although no significant differences were detected, these results are valuable as they demonstrate the stability of muscle lactate and glycogen content under varying GAA concentrations. This consistency suggests that GAA supplementation does not negatively affect energy metabolism in the pectoral muscles of broilers, even at higher doses (e.g., 1200 mg/kg). These findings provide important insights into the physiological responses of broilers to GAA, reinforcing its potential as a safe feed additive under the tested conditions.

### 3.6. The Influence of Guanidinoacetic Acid on Intestinal Morphology in Yellow-Feather Broilers

**Figure 4:**
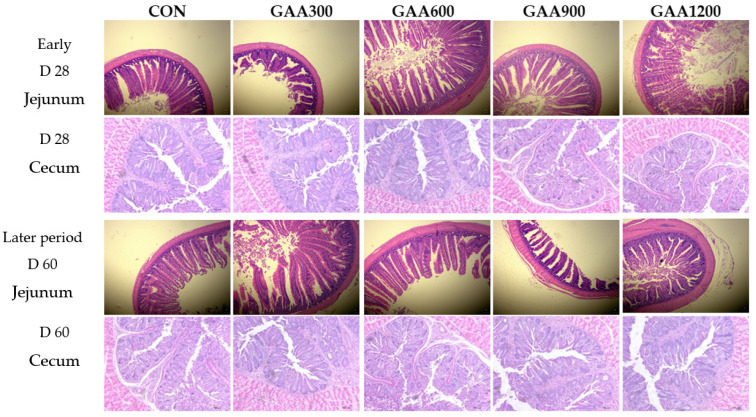
GAA dose effects on the intestinal morphology in yellow-feathered broilers (μm). Note: jejunum section observed under 40× magnification; cecum section observed under 10× magnification.

As shown in Table 5 and Figure 4, this study investigated the effects of dietary guanidinoacetic acid (GAA) supplementation on the intestinal morphology of yellow-feathered broilers across different growth stages. Our findings indicate that GAA supplementation, particularly at higher doses (900 mg/kg and 1200 mg/kg), positively influences jejunal morphology during the early growth phase by reducing crypt depth and increasing the villus-to-crypt (V/C) ratio. These changes suggest improved nutrient absorption capacity and efficient intestinal function, supporting enhanced growth performance in the early stages. However, by the later growth phase (60 days), the effects of GAA on intestinal morphology were not sustained, indicating that its influence may be more significant during the critical early development periods. Additionally, GAA supplementation did not affect cecal morphology throughout the study, highlighting a localized effect on the jejunum. Overall, these results underscore the potential of GAA as a beneficial feed additive that can improve intestinal health and growth performance in broilers during crucial early growth stages. Future research should explore the long-term impacts of guanidinoacetic acid (GAA) and its potential mechanisms of action to further elucidate its role in poultry nutrition.

### 3.7. The Impact of Guanidinoacetic Acid on Muscle Fiber Characteristics of Yellow-Feathered Broilers

As depicted in Table 6, this study evaluated the effects of dietary guanidinoacetic acid (GAA) supplementation on the muscle fiber characteristics of yellow-feathered broilers. The results demonstrated that GAA supplementation did not significantly alter muscle fiber density or diameter in the pectoral muscles during either the early or later growth stages (*p* > 0.05). While no statistically significant differences were observed, there was a slight increase in muscle fiber diameter with higher GAA supplementation levels during the early growth stage. Similarly, during the later growth stage, muscle fiber density showed minor variations among groups, but these changes were not consistent or significant under the conditions of this study. These findings suggest that guanidinoacetic acid (GAA) supplementation may have limited effects on muscle fiber traits in yellow-feathered broilers. Future studies should consider exploring alternative experimental conditions, such as different GAA dosages, feeding periods, or broiler strains, to better understand its potential role in muscle development.

## 4. Discussion

### 4.1. The Impact of GAA on the Growth Performance of Yellow-Feathered Broilers

Guanidinoacetic acid (GAA) is acknowledged and extensively used as a feed additive in many parts of the world, such as the European Union, the United States, and China. Numerous studies have reported on the effects of GAA on growth performance. Ringel and Lemme, along with other researchers, discovered that incorporating 0.6 to 1.2 g/kg of GAA into AA broiler diets significantly improves growth performance and breast muscle yield [10,11,12,13,29]. Including 600 mg/kg of GAA in the diet greatly enhances the average daily gain and feed conversion ratio (FCR) of broilers, while also lowering their mortality rates [30]. Research by Mousavi et al. [14] showed that the addition of 0.6 g/kg of GAA to two energy-level diets enhanced the feed conversion ratio, suggesting that GAA minimizes the energy needed for weight gain. Fosoul’s research further demonstrated that GAA supplementation in low-energy diets improves broiler growth performance. These results collectively affirm the positive influence of GAA on growth performance [15].

In our experiment, the inclusion of varying doses of GAA in the diets did not significantly alter feed intake compared to the control group throughout the trial (*p* > 0.05), aligning with the results of Ahmadipour et al. [31]. Ahmadipour et al. posited that phosphocreatine, a phosphorylated derivative of creatine, acts as a high-energy phosphate reserve in avian species, facilitating rapid energy response. As a direct precursor of creatine, GAA improves the feed conversion rate without significantly changing feed intake, likely due to enhanced energy utilization efficiency. Alternatively, GAA might promote the synthesis of growth-enhancing polyamines such as gamma-aminobutyric acid (GABA), spermidine, and spermine [31]. Smith observed that these polyamines play an anabolic role in the synthesis of DNA, RNA, and proteins [32].

Contrary to other studies, our experiment revealed no significant differences in weight gain or FCR among all test groups compared to the control group. However, the groups receiving GAA300, GAA600, GAA900, and GAA1200 exhibited a 13.01%, 8.97%, 12.95%, and 11.35% increase in average daily gain, respectively, at 28 days of age compared to the control group. Additionally, the FCR for the GAA300, GAA600, and GAA900 groups decreased by 1.90%, 4.74%, and 4.27%, respectively, compared to the control group. These results are in line with the findings of Nasiroleslami et al. and Zhang L. et al. [33,34].

Several physiological and environmental factors may elucidate why certain parameters, such as growth performance and microbiota diversity, exhibited non-significant changes in our study. Nutritional Sufficiency: One key factor could be that the levels of arginine supplied in the diets may have been adequate to meet the growth requirements of the broilers. This sufficiency could diminish the additive effects of GAA on growth performance, as the broilers may not have experienced stress or a nutrient deficit that would typically prompt the body to utilize GAA more effectively. This aligns with the observations by Lemme et al. [35], where an increase in dietary arginine led to a decrease in GAA digestibility, indicating that when nutrients are plentiful, other factors may play a more dominant role in growth than the supplementation of GAA. Microbiota Influence: The relative stability in microbiota diversity across treatment groups might reflect a balanced gut ecosystem where the existing microbial population effectively utilizes available nutrients, regardless of GAA supplementation. Factors such as the initial health status of the birds, housing conditions, and feed quality might have contributed to this observation. Additionally, variations in individual responses to dietary supplements are common in avian species, which may mask any potential improvements attributed to GAA. Adaptation Period: It is also possible that an insufficient adaptation period for the birds to acclimatize to the new diet with GAA contributed to the lack of observed differences in growth performance. The physiological adaptations necessary to leverage the benefits of GAA may take longer to manifest, especially in a dynamic biological system such as the gut microbiota. Environmental Conditions: External factors, such as ambient temperature, humidity, and stress levels during the experimental period, could also play significant roles. In particular, environmental stressors can skew results by impacting feed intake and the efficiency of nutrient utilization, ultimately affecting growth metrics and microbiota composition. The primary reason for the outcomes observed in our study might be that the supplemented arginine sufficiently meets the growth requirements of the broilers, thereby reducing the conversion efficiency of ingested GAA. This phenomenon parallels the additive effect of GAA and arginine content mentioned by Lemme et al. [35]. Lemme et al. found that in arginine-deficient conditions, a diet containing 0.6 g/kg of GAA is entirely utilized, but when the GAA concentration is increased to 1.2 g/kg, the digestibility drops to 86%. Furthermore, when 1.6 g/kg of arginine is included in the diet, the digestibility of GAA in the 0.6 g/kg GAA group decreases to 90%, and in the 1.2 g/kg GAA group, it falls to 70% [35]. Similarly, Tossenberger et al. reported that the utilization rate of GAA is 76.2% when 0.6 g/kg is added to the diet, but it decreases to 45.6% when the additive dosage increases to 6 g/kg. These studies collectively suggest that GAA does not universally enhance growth performance under all conditions [36]. They also indicate that GAA improves broiler weight gain and FCR by enhancing energy utilization efficiency rather than promoting the synthesis of growth-enhancing polyamines, consistent with the conclusions of Mousavi and Fosoul [14,15].

### 4.2. The Impact of GAA on the Slaughter Performance and Meat Quality of Yellow-Feathered Broilers

As the global population increases, consumer standards for chicken breeding have evolved. The focus has shifted from the visual traits of live chickens in the 1930s to carcass characteristics in the mid-20th century, and more recently to the quality and yield of breast meat. Consequently, meat quality has garnered increasing attention from both consumers and producers alike. Reports regarding the effects of GAA on meat quality primarily focus on its role in reducing the incidence and severity of Wooden Breast (WB) [25,37]. However, information on its impact on white striping and spaghetti meat remains relatively sparse, indicating a necessity for further investigation. Numerous studies have reported inconsistent results regarding the impact of GAA on slaughter performance. Metwally et al. suggested that GAA supplementation may increase carcass yield [38]. Conversely, Jiang Tao et al. found that the addition of GAA in the diets of AA broilers could enhance slaughter rate, breast muscle yield, thigh muscle yield, and overall carcass yield, along with a trend toward reduced abdominal fat [17]. In contrast, Majdeddin reported no significant effects on carcass characteristics when adding 0.06% and 0.12% GAA to the diets of Ross 308 roosters [39]. Our study results align with this observation, indicating that varying concentrations of GAA added to the diets of medium-growing yellow-feathered broilers did not exert significant effects on slaughter performance compared to the control group. Crucially, our findings indicate that the drip loss and pH at 45 min post-mortem were significantly improved in the GAA treatment groups compared to the control, corroborating the results of Michiels et al. [40]. ATP, as the primary energy source for cellular functions, plays a pivotal role in muscle metabolism. There are three main energy systems involved in ATP production within muscle cells: (1) aerobic glycolysis, yielding 36–38 moles of ATP per mole of glucose; (2) anaerobic glycolysis generating lactate, which yields 2 moles of ATP per mole of glucose; and (3) the creatine/phosphocreatine system functioning as an energy shuttle. These pathways come into play in scenarios where ATP levels drop below critical thresholds [9,41,42]. When ATP concentration falls below a threshold, the synthesis of phosphocreatine is initiated, leading to the transfer of high-energy phosphate groups to ADP to regenerate ATP [41,42]. As phosphate reserves become depleted, anaerobic glycolysis becomes the primary energy source [43,44]. By incorporating GAA into the diet, the creatine content in muscle tissue is significantly increased, which consequently delays glycolytic processes and reduces post-mortem lactate production, thereby elevating pH at 45 min post-mortem. This mechanism may also help mitigate protein denaturation, enhancing the interactions between proteins and water, ultimately improving meat quality and reducing drip loss. Similar outcomes have been reported by Zhang L. et al. [34].

### 4.3. The Impact of GAA on Serum Biochemical Parameters in Yellow-Feathered Broilers

Zhang reported that the levels of certain serum components may be influenced by dietary GAA, depending on the dosage and feed composition [16]. DeGroot found that the addition of GAA to diets had no significant effects on blood cell counts (including white blood cells, heterophils, lymphocytes, monocytes, eosinophils, and basophils), but the proportions of heterophils and lymphocytes were impacted [45]. These findings suggest that GAA may exhibit regulatory effects under specific physiological states, warranting further investigation into its influence on various immune parameters. Despite this, Tossenberger et al. reported that even with GAA amounts reaching 0.6% in the diet, major serum biochemical indices (including serum protein, cholesterol, glucose, and others) remained largely unchanged, indicating a possible modulation of metabolic processes rather than straightforward nutritional supplementation [36]. Michiels et al. observed no significant differences in serum glucose and uric acid levels in broilers fed corn-based diets without fish meal [40]. Notably, Abasht et al. reported that samples affected by WB exhibited a significantly higher content of the γ-glutamyl amino acid metabolic byproduct 5-oxoproline, approximately 1.57 times that of unaffected samples [46]. In our investigation, only the ALT levels showed significant differences among groups. As a sensitive indicator of liver damage, ALT concentrations increase rapidly following liver cell breakdown, emphasizing the need to monitor liver function with GAA supplementation. We observed a significant elevation in ALT levels in the 1200 mg/kg GAA group, a phenomenon that may be closely related to metabolic stress on the liver. We believe this result underscores the importance of further investigating the effects of GAA on liver health. Future research should focus on conducting longer-term assessments, incorporating histological analyses and additional biochemical markers, to comprehensively elucidate the impact of GAA on liver integrity while promoting meat quality. This observation not only provides direction for optimizing dietary GAA inclusion but also emphasizes the need for caution in the use of additives in production practices to ensure that benefits can be maximized while mitigating potential health risks.

Other serum biochemical parameters did not exhibit significant differences. Therefore, it can be hypothesized that dietary GAA supplementation may mitigate the severity and incidence of WB to some extent, resulting in no significant changes in other serum biochemical indices.

### 4.4. The Influence of GAA on Pectoral Muscle in Yellow-Feathered Broilers

Currently, the primary pathological conditions affecting chicken breast muscle include white striping (WS), spaghetti meat (SM), and WB, all of which significantly degrade meat quality. WB is particularly prominent and is characterized by increased hardness of the pectoralis major muscle, often associated with pale coloration, higher drip loss, cooking loss, and shear force. Sihvo et al. have documented the morphological changes induced by WB, such as discoloration, surface mucus, and alterations in hardness [47]. While existing research indicates that GAA can mitigate the incidence and severity of WB, the variability in findings across different studies highlights the pressing need for further investigation into its efficacy under varying production conditions [25,37]. Kuttappan et al. suggested that WB is more commonly found in faster-growing and heavier broilers, which likely results from breast muscle growth mechanisms that induce hypertrophy of existing fibers alongside minor fiber hyperplasia, thereby impacting fiber diameter and density [48]. In this study, no significant differences were detected in the fiber diameter or density of the breast muscle between the GAA treatment groups and the control group. Nonetheless, as a precursor to creatine, GAA provides a beneficial energy supply that may delay the onset of glycolysis and reduce lactate accumulation, aligning with our findings. Although no significant differences were observed in lactate levels in muscle tissue, a trend toward decreased lactate levels was noted across all treatment groups, while the glycogen content in the breast muscle did not exhibit significant differences. This suggests that GAA may promote a more efficient energy metabolism in muscle tissue, which could be pivotal for enhancing overall production performance.

### 4.5. The Impact of GAA on the Intestinal Morphology of Yellow-Feathered Broiler Chickens

The intestine serves as the primary site for nutrient absorption, with critical indicators including villus height, crypt depth, and their ratio (V/C ratio) reflecting the small intestine’s digestive and absorptive capabilities. Taller intestinal villi significantly increase the surface area available for nutrient absorption, correlating with improved absorption efficiency. Shallow crypts, indicating a higher abundance of mature epithelial cells, also enhance nutrient absorption effectiveness. An increased V/C ratio typically signifies superior nutrient absorption capabilities [49].

The structure of the intestinal mucosa, characterized by tiny villi formed from epithelial cells and the underlying lamina propria, varies in morphology among species, ages, and intestinal regions [50]. These villi optimize the mucosal surface area, promoting digestive functions. Notably, an increase in villus height demonstrates enhanced digestive and absorptive functions within the intestine. A reduction in crypt depth can reflect decreased cell generation rates and increased epithelial maturity, ultimately enhancing nutrient absorption [51].

The functionality of the small intestine is paramount, with the V/C ratio serving as a key indicator of its efficiency. An elevated V/C ratio denotes enhanced digestive and absorptive performance, aligning with previous studies that establish the importance of intestinal morphology in nutrient absorption [52].

The gastrointestinal tract remains a metabolically active system requiring substantial oxygen [53]. In inflammatory states, intestinal epithelial cells may experience hypoxia [54], making creatine vital for promoting epithelial repair and alleviating mucosal inflammation through improved cellular energy [55]. Dietary supplementation with GAA has been shown to enhance cellular energy metabolism by increasing levels of creatine and phosphocreatine alongside ATP production, facilitating cellular proliferation and boosting protein synthesis [12].

In our study, GAA 900 and GAA 1200 treatments significantly reduced jejunal crypt depth compared to the control group, while groups GAA 600, GAA 900, and GAA 1200 exhibited significantly increased jejunal villus-to-crypt ratios. The reduced crypt depth indicates a decrease in the rate of cell production while improving epithelial cell maturity. This finding is consistent with the conclusion by Glover that GAA supplementation may promote intestinal epithelial repair and ameliorate mucosal inflammation, reinforcing the hypothesis that GAA enhances intestinal absorption efficiency through improved cellular mechanisms [55]. Furthermore, the increased villus-to-crypt ratio suggests an enhancement in the overall absorptive capacity of the intestine, supporting the notion that GAA may act to optimize energy utilization and improve growth rates in broilers.

Incorporating GAA into feed may alleviate oxidative stress on the intestinal epithelium by promoting epithelial cell repair, reducing crypt cell differentiation, and subsequently decreasing crypt depth. Such improvements appear to enhance the intestinal barrier function and nutrient absorption capability, leading to enhanced production performance.

### 4.6. Potential Implications of GAA Supplementation for Livestock Health and Disease Management

Although this study focused on the effects of guanidinoacetic acid (GAA) supplementation on growth performance, meat quality, and intestinal morphology in yellow-feathered broilers, the findings regarding improved energy metabolism and enhanced physiological status may have broader implications for livestock health management. In particular, improved nutrient absorption and reduced metabolic stress, as observed here, could support stronger immune responses and better resilience to infections. While porcine reproductive and respiratory syndrome virus (PRRSV) primarily affects swine, the principles of optimizing animal nutrition to bolster immune function are universally relevant across species. Therefore, the positive effects of GAA seen in broilers suggest potential benefits if applied in swine production, especially in regions like Northeast India where PRRSV remains a significant challenge. Nutritional strategies incorporating GAA may enhance vaccine responsiveness and reduce disease severity by improving host energy status and immune competence. We recommend further species-specific investigations to evaluate GAA’s role in swine health and PRRSV prevention, which could inform integrated management approaches combining vaccination and nutritional support.

## 5. Conclusions

This study suggests that dietary supplementation with guanidinoacetic acid (GAA) at levels of 300 mg/kg, 600 mg/kg, and 900 mg/kg may enhance early-stage growth performance in yellow-feathered broilers. Although the improvements in average daily weight gain were not statistically significant (*p* > 0.05), a trend was observed that warrants further investigation. Additionally, these dosages significantly improved post-slaughter pH levels in breast muscle, contributing to better meat quality, thereby highlighting the potential positive role of GAA in meat production. However, at the highest dosage of 1200 mg/kg, there was a notable increase of 53.47% in serum ALT levels, indicating potential hepatic stress (*p* > 0.05). This finding underscores the necessity of caution when considering higher GAA dosages to avoid negative impacts on liver function. Furthermore, supplementation at 900 mg/kg and 1200 mg/kg significantly reduced crypt depth and increased the villus-to-crypt ratio in the early jejunum (*p* < 0.01), thereby enhancing intestinal barrier function. In light of these findings, a dosage of 900 mg/kg of GAA is recommended as optimal for promoting growth performance and meat quality, while being cautious of elevated doses that may impact liver health. Continued research is essential to fully understand the long-term implications of GAA supplementation in poultry diets, particularly concerning liver function and overall health.

## Figures and Tables

**Table 1:** Composition and nutritional levels of basal diets (air-dried basis, %).

Item	Early (1~28 d)	Later Period (29~60 d)
Ingredient		
Corn	58.8	62.5
Soybean meal	32.6	28.6
Wheat bran	0.6	0.9
Soybean oil	1.7	3.0
Corn gluten meal	2.3	1.0
Premix ^(1)^	4.0	4.0
Total	100	100
Nutrient levels ^(2)^		
ME/(MJ/kg)	12.14	12.52
CP	20.73	18.58
Arg	1.20	1.00
Lys	1.15	1.03
Met	0.46	0.41
Ca	0.98	0.89
Total phosphorus	0.65	0.61

^(1)^ The premix feed addition indicated in Table 1 is 4.0%, which means that 40 kg is added per ton of feed. It is noteworthy that the contents of vitamins and minerals listed in the footnote are within safe levels and do not exceed toxic limits. The premix was provided with VA 12,000 IU, VD 32,500 IU, VE 20.0 mg, VK 33.0 mg, VB1 3.0 mg, VB2 8.0 mg, VB6 7.0 mg, VB12 0.03 mg, pantothenic acid 20.0 mg, nicotinic acid 50.0 mg, biotin 0.1 mg, folic acid 1.5 mg, Fe 96 mg, Cu 25 mg, I 0.9 mg, Zn 98 mg, Mn 105.4 mg, and Se 0.04 mg. ^(2)^ The nutrition level is the calculated value.

**Table 2:** Composition of the premix added to the diets (air-dried basis, %).

Ingredients	Early (1~28 d)	Later Period (29~60 d)
Limestone	4.0	4.7
Chicken feather meal	0.35	0.3
Trace mineral premix	1.0	1.0
Calcium carbonate	12.0	11.1
Stone powder	14.0	12.8
Antioxidant ^(1)^	0.1	0.1
Salt	3.0	3.0
L-Arginine (98%)	1.15	1.0
DL-Methionine	1.4	1.2
Corn starch	2.0	4.0
Choline chloride	1.0	0.8
Total	40.0	40.0

^(1)^ The antioxidant used in the study was sourced from Jiangxi Yuanchang Industrial Co., Ltd., Ningzhou Industrial Park, Xiushui County, Jiangxi Province, China. The product contains key components such as EMQ and enhancers.

**Table 3:** Effects of different doses of guanidinoacetic acid on serum biochemical parameters of yellow-feathered broiler chickens during the early (28 Day) and later (60 Day) growth periods.

Item	CON	GAA 300	GAA 600	GAA 900	GAA 1200	SEM	*p*		
							*p*	*L*	*Q*
Early (28 Day)									
ALT, U/L	2.33	2.33	2.50	2.67	2.17	0.17	0.923	0.966	0.966
GLU, mmol/L	15.57	14.73	14.27	13.68	16.00	0.31	0.111	0.722	0.035
TG, mmol/L	0.46	0.53	0.49	0.42	0.43	0.02	0.630	0.439	0.304
HDL, mmol/L	2.22	2.10	2.13	2.20	1.97	0.04	0.397	0.185	0.743
LDL, mmol/L	1.06	0.96	0.94	1.04	0.95	0.05	0.925	0.647	0.715
Later period (60 Day)									
ALT, U/L	2.17 ^ab^	1.83 ^b^	1.83 ^b^	3.00 ^ab^	3.33 ^a^	0.21	0.048	0.026	0.048
GLU, mmol/L	14.50	14.20	14.65	15.02	15.75	0.24	0.283	0.078	0.180
TG, mmol/L	0.41	0.57	0.53	0.41	0.47	0.03	0.217	0.878	0.109
HDL, mmol/L	1.94	2.07	2.01	1.98	2.13	0.44	0.712	0.357	0.924
LDL, mmol/L	1.12	0.99	1.28	0.94	1.12	0.05	0.155	0.820	0.960

In the same row, values with no letter or the same letter superscripts mean no significant difference (*p* > 0.05). while with different letter superscripts mean significant difference (*p* < 0.05). The same as below.

**Table 4:** Effects of different doses of GAA on muscle lactic acid and glycogen in yellow-feathered broilers.

Item	CON	GAA 300	GAA 600	GAA 900	GAA 1200	SEM	*p*		
							*p*	*L*	*Q*
Early (28 Day)									
LD, mmol/g	0.60	0.77	0.56	0.59	0.83	0.05	0.232	0.288	0.307
GLU, mg/g	0.95	0.87	1.12	0.57	0.90	0.09	0.451	0.641	0.774
Later period (60 Day)									
LD, mmol/g	3.05	2.51	3.03	2.82	2.41	0.19	0.786	0.488	0.848
GLU, mg/g	0.75	0.74	0.74	0.79	0.66	0.05	0.948	0.765	0.694

**Table 5:** Effects of different doses of GAA on the intestinal morphology of yellow-feathered broilers, μm.

Item	CON	GAA 300	GAA 600	GAA 900	GAA 1200	SEM	*p*		
							*p*	*L*	*Q*
Early (28 Day)									
Jejunal villus height	1274.81	1189.84	1288.56	1317.81	1104.77	36.39	0.355	0.425	0.421
Jejunal crypt depth	123.92 ^a^	139.5 ^a^	114.74 ^ab^	95.56 ^bc^	85.04 ^c^	5.14	<0.01	<0.01	0.004
V/C	10.35 ^cd^	8.54 ^d^	11.25 ^bc^	13.92 ^a^	13.09 ^ab^	0.52	<0.01	<0.01	0.041
Cecal crypt depth	282.74	287.06	264.81	318.94	295.70	8.56	0.387	0.337	0.632
Later period (60 Day)									
Jejunal villus height	1373.31	1626.04	1347.26	1434.87	1327.16	47.31	0.324	0.603	0.226
Jejunal crypt depth	89.41	98.63	90.79	78.16	77.94	2.77	0.083	0.049	0.101
V/C	15.39	16.76	15.23	18.38	17.44	0.66	0.518	0.228	0.859
Cecal crypt depth	120.11	115.20	125.64	113.10	114.54	5.14	0.962	0.726	0.919

Note: Within the same row, data groups marked with different superscript letters (e.g., a, b, c, d) indicate statistically significant differences at *p* < 0.05; groups sharing the same letter or without letters indicate no statistically significant difference (*p* > 0.05).

**Table 6:** Effects of different doses of GAA on chest muscle fibers in yellow-feathered broilers.

Item	CON	GAA 300	GAA 600	GAA 900	GAA 1200	SEM	*p*		
							*P*	*L*	*Q*
Early (28 Day)									
Density of muscle fiber/mm^2^	920.07	934.93	924.76	941.13	905.85	7.08	0.587	0.787	0.237
Diameter of muscle fiber/μm	33.00	33.84	35.34	34.92	35.41	0.49	0.463	0.089	0.750
Later period (60 Day)									
Density of muscle fiber/mm^2^	470.31	454.50	432.42	439.96	463.79	5.77	0.193	0.346	0.056
Diameter of muscle fiber/μm	53.55	54.26	51.57	53.78	49.27	0.84	0.319	0.170	0.332

## Data Availability

The original contributions presented in this study are included in the article. Further inquiries can be directed to the corresponding authors.
